# Nutritional Status in Peritoneal Dialysis: Nutritional Guidelines, Adequacy and the Management of Malnutrition

**DOI:** 10.3390/nu12061715

**Published:** 2020-06-08

**Authors:** Thomas Kiebalo, Jacqueline Holotka, Ireneusz Habura, Krzysztof Pawlaczyk

**Affiliations:** 1 Department of Nephrology, Transplantology and Internal Medicine, Poznan University of Medical Sciences, 60-355 Poznan, Poland; s74581@student.ump.edu.pl (T.K.); jacquelineholotka@gmail.com (J.H.); 2 Department of Nephrology, University Hospital of Karol Marcinkowski in Zielona Gora, 65-046 Zielona Gora, Poland; irha@tlen.pl

**Keywords:** peritoneal dialysis, nutritional status, adequacy, protein intake, renal replacement therapy

## Abstract

The positive impact of nutritional status on the health and treatment adequacy of peritoneal dialyzed patients has been well established. Protein intake is an important factor used to stratify malnutrition, with inadequate intake leading to protein-energy wasting during the course of therapy. In this review, we discuss the recommendations made by nephrological societies regarding nutrition in this population of dialysis patients. Special attention is given to the intake of protein, and recommendations on the intake of micronutrients are also discussed. Furthermore, factors that may impair nutritional intake and balance are discussed, with mention of the innovative strategies utilized to combat them. In light of inconsistent recommendations that vary between each respective society, as well as a general lack of concise information, it is our intention to call for further research regarding nutritional recommendations in peritoneal dialysis (PD), as well as to advocate for clear and accessible information for patients.

## 1. Introduction

Peritoneal dialysis (PD) is one of the two main modalities of renal replacement therapy (RRT) offered to patients with end-stage renal disease (ESRD). This therapy involves solute and fluid exchange over the peritoneal membrane, allowing for the efficient elimination of waste products. Implementation of such therapy has been associated with various long-term pathophysiologic changes which result in adverse health consequences. One of these is malnutrition, occurring in 30–50% of patients [1]. A new paradigm in PD care is being established, placing the patient at the center of a multidisciplinary team. High-quality PD requires cooperation between the care team and the patient. Various parameters for administering high-quality PD have been established, one of which is Nutritional Status. High-quality PD guidelines recommend the regular and thorough evaluation of patient appetite, body weight measurements, clinical status as well as dietary intake and laboratory markers of nutrition (albumin, potassium, bicarbonate and phosphate). Despite this official recommendation, specific dietary guidelines have not yet been made clear.

Dietary recommendations are not universal and moreover are influenced by numerous factors. Standard of care currently suggests 1.2–1.3 g/kg of protein per day, but with 41–42% of dialysis patients considered clinically malnourished, this is an amount that is clearly not being met [2]. Patients have been reported to consume lower amounts of dietary protein when switching from hemodialysis (HD) to PD therapy, and this may be a consequence of the comparatively higher levels of uremic toxins present, known to be appetite suppressants [3]. Mortality in PD patients may be estimated by measuring total body nitrogen, a surrogate marker for protein intake [4]. Furthermore, older age, chronic inflammation and loss of residual renal function all contribute to both inadequate protein intake [2] and altered protein balance PD patients have been shown to benefit from dietary interventions in the form of protein supplementation with improvement in albumin levels, muscle strength, caloric intake and quality of life [3].

## 2. Epidemiology

An estimated 272,000 individuals around the world today are receiving PD [5], representing 11% of all patients receiving RRT [5]. Global trends are displaying an increased use of PD in countries such as China, Thailand and the US. Asia recognizes the economic benefits of PD, which is less costly compared to HD, while US trends are largely due to changes in RRT funding after epidemiological studies showed improved 5-year survival of PD compared to HD [5]. In contrast, Europe and Canada have seen a general decline in patients receiving PD [5]. In Europe, the overall percentage of PD patients is decreasing due to increased rates of transplantation and HD treatment. Similarly, in Canada, the ability to provide high-quality HD has improved, leading to fewer PD patients. Besides medical and economic factors that affect the type of RRT selected, psychology, education and patient beliefs and values also contribute to these epidemiological changes [5,6]. Lameire et al., go on to state that patients should be offered both PD and HD therapy in a non-biased way [6]. As the popularity of PD continues to grow, it is important to discuss and analyze current nutritional guidelines in order to expose any drawbacks or barriers and help to improve quality-of-life as well as to reduce morbidity and mortality in PD patients. Guidelines from international societies are presented in Table 1 and Table 2.

## 3. Significance of Nutritional Status in PD

Patients undergoing HD have been reported to have greater muscle mass and mid-arm circumference than those undergoing PD therapy [10]. PD patients are also susceptible to micronutrient (vitamin and mineral) deficiencies as a consequence of abnormalities in metabolism, intestinal dysfunction and inadequate nutritional intake [11]. These deficiencies may exacerbate existing comorbidities including cardiovascular disease, sexual dysfunction, anemia [12] and sarcopenia [13]. Traditional PD solutions are high in glucose and provide around 300–450 kcal per day. This attribute may be undesirable for overweight patients as well as those suffering from diabetes mellitus [4], who are known to store this form of energy as adipose tissue [14]. This has led to the development of new dialysis solutions, such as those that replace the osmotic effects of glucose with icodextrin or amino acids [15]. The use of amino acid (AA) containing dialysis solutions may be of benefit to patients, particularly to those with low daily protein intake (DPI) [16,17]. The benefit of a combined AA and glucose dialysate solution for patients is the ability to simultaneously provide both protein and calories [17,18]. Supplementing AA in the dialysate has been shown to enhance anabolic status, by increasing nitrogen balance as well as improving total protein concentration [19]. However, as Brulez et al., mention, the desirable effects of AA based PD dialysate solutions, mainly improved nutritional status and lipid profile, must be weighed against the potential to promote cardiovascular disease due to elevated plasma homocysteine levels [20], while also being aware of the potential for elevations in the plasma urea concentration [20]. On the other hand, solutions high in glucose may be of value for individuals who are already malnourished [4], as a result of the cumulative effects of PD-associated pathophysiological changes. In addition to polypharmacy—typical in ESRD patients—these factors all contribute to longer hospital stays, increased medical costs and poor health outcomes [10]. 

**Figure 1:**
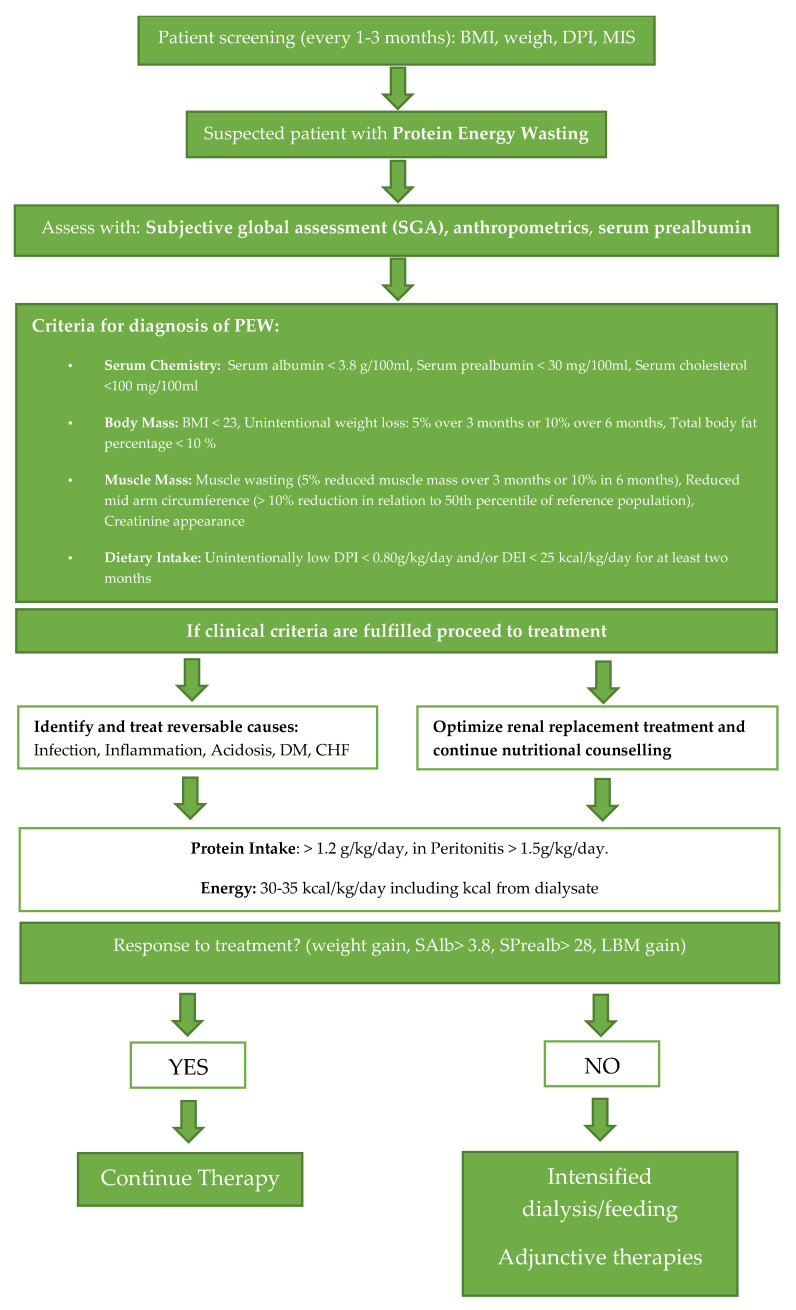
Assessment of nutritional status in a PD patient.

Adequate nutrition has been deemed vital to the success of both peritoneal and hemodialysis [21]. In fact, Schmiker maintains that in order to ensure the success of long-term dialysis treatment, patients should enter treatment in an anabolic state, and the nutritional needs of such patients (protein consumption, vitamin intake, fluid restriction, energy supply) should all be addressed in order to prevent a catabolic state [21]. It has been suggested that traditional PD solutions (low pH and high glucose) modify the integrity of the peritoneal membrane [22]. Further alterations to the filtration membrane may be a consequence of uremia and the presence of the peritoneal catheter [22], and cumulatively, these changes promote a high transport state. “Ultrafiltrators” lose high levels of protein in the dialysis solution, leading to hypoalbuminemia [23]. Additionally, many patients treated with dialysis suffer from protein-energy wasting (PEW) as a result of inflammation, chronic acidosis, insufficient nutrient intake and the hypercatabolic nature of their illness [24]. PEW characteristics were standardized in 2008 by the International Society for Renal Nutrition and Metabolism, which divided PEW into further groupings, mainly: abnormal serum biochemistry, reduced body mass, reduced muscle mass and unintentional low dietary intake [25]. Weight loss, an indicator of malnutrition, has been reported in patients undergoing PD therapy despite increases in caloric intake and unaltered activity levels [26]. An observational study conducted by Dong et al., assessed the impact of PEW on survival. In 305 patients receiving PD, those with a DPI of <0.73 g/kg/day had worse outcomes, and it was only until a DPI of >0.94 g/kg/day was achieved that patients experienced better outcomes and fewer complications such as peritonitis [27]. Recognizing PEW has been critical for proper assessment of nutritional status, and nutritional indexes and scores have been developed for this very purpose. Tools such as the Subjective Global Assessment (SGA), body anthropometry and body composition analysis help in the diagnosis [28]. When PEW is recognized, it is critical to undertake comprehensive management especially to ensure optimal dialysis, correction of any pH disturbances and incorporation of dietary supplementation [28]. The stepwise evaluation illustrated in Figure 1. provides a concise schematic for how to diagnose and manage patients with PEW. For patients meeting the diagnostic criteria, treatment with protein supplementation or further adjunctive and intensified therapies can be perused.

Any factors impairing nutritional intake should be addressed. It is especially critical to assess the presence of metabolic acidosis: The correction of this derangement has proven to be effective in improving the nutritional status of PD patients [29]. Notably, therapy with oral sodium bicarbonate successfully improves plasma bicarbonate as a marker of acid-base balance versus placebo (*p* = 0.002) [29]. Oral sodium bicarbonate therapy resulted in a higher SGA score compared to placebo (*p* = 0.0003). In patients with initially low bicarbonate levels, supplementation of oral sodium bicarbonate can also help to reduce the rate of renal decline and prevent the subsequent development of ESRD [30] As described by Tian et al., PD patients with metabolic acidosis can be further classified into groups with and without anion gap [31]. Differences exist between these two groups; for example, patients who presented with anion gap metabolic acidosis had lower DPI values as well as higher serum levels of phosphate and urea [31]. Furthermore, the authors conclude that patients on continuous ambulatory PD with relatively better RRF were at higher risk of developing non-anion gap metabolic acidosis, alluding to the fact that the development of acidemia may be a result of renal bicarbonate loss [31]. Failure to recognize the presence of acidosis results in the gradual break down of skeletal muscles occurring due to the activation of the ubiquitin-proteasome machinery [32].

The uremic syndrome should be considered as a potentially fatal interaction among inflammation, malnutrition, low levels of albumin in the plasma, accumulated protein-bound solutes and generation of non-nutritionally related toxins. Malnutrition linked to tissue breakdown results in the release of toxic compounds, thus creating a vicious cycle. Tissue degradation results in the release of acid, purines, phosphate, potassium, β2-microglobulin, and myoglobin, all of which have been shown to exert biologic action [33]. Not only optimal dialysis but also optimal nutritional intake and optimal utilization of these nutrients should help neutralize this chain of events [33]. In patients on PD with a comparable weekly removal of small uremic toxins as in HD patients, the removal of middle molecules will be superior [34]. The comparatively better removal of protein-bound molecules might also explain the slower decline in residual renal function found in PD patients [34]. Interestingly, one way to lower uremic toxin is by administering probiotics [35].

## 4. Biochemical and Clinical Markers of Malnutrition

There are several accepted strategies for stratifying malnutrition in PD. One marker used to assess nutritional status is serum albumin. The usefulness of this marker is due to the fact that its level is influenced by DPI [36,37,38,39]. Furthermore, it is low in cost and easily accessible [40]. However, its use as a diagnostic marker is limited as albumin levels may be influenced by many factors such as liver pathology and gastrointestinal and renal losses [41]. Albumin levels are also decreased in inflammatory states characterized by high TNF-alpha and IL-6 [38], and their preferred use is as a screening tool in clinical practice [41]. Another useful tool for comprehensive assessment of nutritional status is the subjective global assessment (SGA). The analyzed parameters include components of medical history and physical examination [42,43]. The former takes into account dietary intake, changes in weight, gastrointestinal complaints, functional capacity and nutritional requirements of the medical history, while physical findings are concerned with signs of fat and muscle wasting, as well as fluid balance alterations resulting from inadequate nutrition [42]. The strength of this assessment tool is also its low cost, and another desirable trait is that it can be administered by nurses and dieticians as part of a multidisciplinary renal team [42]. What is more, the SGA is able to detect trends in the nutritional status of patients [44], a trait which allows for a more comprehensive assessment over time versus one time biochemical marker levels [44]. A revised form of the SGA is the Malnutrition-Inflammation score (MIS). Additional parameters in this assessment tool include serum albumin level, total iron binding capacity and body mass index [45,46]. The use of the MIS for assessment of nutritional status has been recommended by KDOQI over the SGA [47]. The MIS recognizes the close association between inflammation and malnutrition in dialysis patients [45]. In addition to biochemical markers of nutritional status, clinical markers are also of significant value when stratifying patients treated with PD. Bioimpedance analysis (BIA) is a means by which researchers can measure lean and adipose body mass and fluid volume content in intracellular and extracellular compartments, important for assessing hydration status [48]. Akbulut et al., report that in addition to being used to manage dry body weight, BIA can be used to evaluate a PD patient’s nutrition [49], and Hoppe et al., state that anthropometric measurements may be used as a complement for the assessment of nutritional status in said patients [48]. Notably, a study by Edefonti et al., successfully evaluated the prevalence of malnutrition in children on PD with the use of both BIA and anthropometry as indices of nutritional status. The authors concluded that BIA is more sensitive that anthropometric measurement for the detection of alterations in body composition [50]. Other valuable clinical markers for routine evaluation of nutritional status in PD patients include a history of weight loss, percentage of standard weight, BMI and clinical evaluation of muscle and subcutaneous fat mass [49].

## 5. Adequacy in Peritoneal Dialysis

Adequacy of dialysis is assumed to be determined solely by the clinical well-being of a patient, though it is evident that other factors such as financial status and a patient’s unique interaction with dialysis procedures and materials also have a powerful impact [51]. Other determinants contributing to adequacy include patient blood pressure and volume status, nutritional status, acid-base status and mineral and bone pathologies. The urea reduction ratio (URR) and Kt/V are two methods employed to determine adequacy in PD. URR represents the percentage reduction in blood urea nitrogen (BUN) following a single treatment of dialysis [52]. This is determined by comparing blood urea values at the beginning of treatment to blood urea values at the end [51]. A similar procedure is conducted when determining Kt/V, where K represents the dialyzer urea clearance (the rate at which blood passes through the dialyzer expressed in milliliters per minute (mL/min)), t is the total treatment time, and V is the total volume within the body over which urea is distributed, with the ratio representing the dose of dialysis used in a single treatment [4]. As Kt/V accounts for the quantity of urea removed with excess fluid, it is considered to be more accurate in the measurement of adequacy compared to URR. Current guidelines define adequacy as weekly measurements of Kt/V equal to or greater than 1.7 [53]. Adequacy targets must encompass the removal of both urea and fluid and must be based solely on removal of urea by means of PD only, disregarding urine and renal clearance entirely [53]. The Kt/V value is an important predictor of mortality in patients on long-term PD, as established by Rocco et al. [54]. When the above-mentioned target values are not successfully met, patients must be evaluated for malnutrition, overhydration and complaints of uremia [53]. The correlation between nutritional status and dialysis adequacy was established by Hemayati et al., for patients suffering from chronic renal failure [55]. The study indicates that alterations in the protein catabolic ratio (PCR), a measure of nutritional status, were significantly and positively associated with both Kt/V and time-averaged concentration of urea (TAC), the two indicators of dialysis adequacy (*p* < 0.001) [55].

## 6. Complications of Malnutrition in PD Patients

Malnutrition complicates PD treatment by directly contributing to the development of infection; in turn, infectious diseases also directly impact upon nutritional intake and adequacy. Infection is a notable source of morbidity in dialyzed patients, with septicemia and other infections accounting for 8% of deaths in US dialysis recipients [56]. Strikingly, recent studies demonstrate that malnourished patients with ESRD have higher rates of infection, poor rehabilitation and mortality amidst other complications [57]. Such complications may be closely linked to protein-energy wasting (PEW), which is commonly seen in CKD patients and is the strongest predictor of morality in this group [58]. PEW hinders immunity in both PD and HD patients, with PD patients displaying comparatively higher risk for infection [59,60]. Studies by van Diepen et al., conclude that the association between PEW and the development of infections in dialysis patients is significant enough to warrant routine screening of nutritional status in all dialysis patients as a means of minimizing infection rates [59,60]. “Exit site infections” are infections surrounding the catheter exit point and are commonly caused by bacteria and fungi [60]. Increased susceptibility to infections in CKD patients may be linked to hypoalbuminemia that results from malnutrition, uremia or the upregulated inflammatory response in dialysis patients [61]. Furthermore, hypoalbuminemia has been associated with septicemia, pneumonia and other inflammatory responses [62]. A study by Ozturk et al., demonstrate that PD patients with declining albumin levels are at a significantly increased risk for development of peritonitis [63]. Prasad et al., report that rates of peritonitis are high in patients on continuous ambulatory peritoneal dialysis (CAPD) and that malnutrition indices can be utilized to predict peritonitis in this cohort [64].

In general, malnutrition contributes to weight loss, impaired immunity and mucosal damage, subsequently resulting in increased risk of pathogen invasion [65]. Infection goes on to exacerbate malnutrition in a vicious cycle [65]. For example, anorexia and poor caloric intake in PD patients is further compounded by the appetite loss typical of acute infectious disease, related to the release of IL-1 during the acute phase response [66]. Results from a 2006 study by Dong et al., identified the development of new systemic infections as one of the most significant risk factors resulting in malnutrition in their cohort of PD patients [27]. PD patients are prone to developing malnutrition-inflammation-atherosclerosis (MIA) syndrome, a complex interaction between malnutrition, high circulating pro-inflammatory cytokines and atherosclerosis in ESRD patients [67]. It has been shown that morbidity and mortality rates in PD patients increase as the syndrome progresses [67]. MIA syndrome has been associated with increased cardiovascular mortality and is responsible for most premature deaths in patients receiving PD [68]. Elevated levels of the inflammatory marker C-reactive protein (CRP) directly correlate to malnutrition, reduced fluid removal and mortality in RRT patients [67]. HD patients have better outcomes than PD patients with regards to the malnutrition component of MIA syndrome; inflammation is comparable for both RRT modalities, and PD proves advantageous for atherosclerosis [67]. Shahab et al., claim a correlation between malnutrition and inflammation that plays a significant role in MIA syndrome pathogenesis [67]. Further, the authors outline that malnutrition is more prevalent in patients starting PD, with malnutrition and inflammation serving as markers for outcomes in said patients [67]. Notably, nutritional support may decrease the prevalence of MIA syndrome in dialyzed patients [67]. Maraj et al., suggest that the alkaline pH of plant-based foods can reduce the production of pro-inflammatory cytokines as well as decrease metabolic acidosis in patients [69]. Their 2018 article proposed that recurrent guidelines focus on increasing antioxidant, phytochemical and fiber intake as a means of improving nutritional and cardiovascular status. It is worth mentioning that in a study by Altieri et al., patients suffering from MIA syndrome were refractory to all conventional modalities of treatment and management; prolonged low-dose steroid treatment was effective in improving both inflammatory symptoms and nutritional status [70].

## 7. Nutritional Recommendations for Pediatric Patients Treated with Peritoneal Dialysis

Children have unique nutritional demands. Inadequate nutritional status in children younger than five years of age results in detrimental loss of development and lasting health disparities [71]. What is more, nutrition plays a vital role in the development of the brain during the postnatal and preschool periods, as neurons undergo rapid development and synaptic pruning during the first five years of life. Unfortunately, children who suffer from CKD are faced with impaired nutritional status directly resulting in sub-optimal growth [72]. This may be attributed to growth hormone and insulin-like growth factor I axis dysregulation, metabolic acidosis, anemia, nutritional deficiencies, renal osteodystrophy and inflammation [72]. Fabio et al., suggest that cachexia or protein-calorie malnutrition is a problem that many PD-treated children encounter and contributes to morbidity and mortality [73]. The multifactorial nature of cachexia mandates that children on PD must undergo a complete history and physical examination in the assessment of their nutritional status, including a thorough assessment of biochemical indices, anthropometry, dietary intake and combined score systems. Their nutritional status should be managed by a multidisciplinary team in order to ensure not only adequate intake of proteins and calories but account for corrections of metabolic control, acidosis, hyperparathyroidism and anemia [73]. In addition to optimal dosing of dialysis, specific drugs including recombinant human growth hormone may be prescribed [73]. Thuc et al., reported in 2019 that children treated by PD showed significant nutritional deficiencies, including low levels of Vitamins A and D, iron and demonstrated low serum albumin [11]. The National Institute of Diabetes and Digestive and Kidney Diseases have outlined nutritional recommendations for pediatric patients. As PD removes a higher amount of protein from the body than HD, larger quantities of protein must be consumed as part of their diet [74]. The National Institute acknowledges that daily sodium intake depends on a variety of factors such as the CKD type in question [74]. Phosphorus and potassium are especially highlighted as these build up in the bodies of CKD children and should be limited with the help of a dietician [74]. Seattle Children’s Hospital recommends limiting the consumption of dairy in pediatric patients on dialysis but maintains that plant-based alternatives should be introduced in order to maintain phosphorus and calcium intake and ensure proper bone development [75]. “Renal vitamins” such as Nephrovite or Nephronex are also recommended during dialysis treatment to compensate for vitamin loss during RRT [75]. Erythropoietin or darbepoetin supplementation may further support erythropoiesis in CKD-burdened children [75]. Nguyen et al., emphasize the importance of individualized management and caution against a “one-size-fits-all” approach to pediatric PD nutrition [76]. Trends in growth should be monitored and nutritional recommendations adjusted accordingly by a physician or dietician. The initial evaluation of the child should assess weight, height and body mass index, and for those aged 36 months or younger, head circumference should be noted [76]. The recommended parameters and frequency of nutritional assessment for children with CKD is outlined by the Kidney Disease Outcomes Quality Initiative (KDOQI) 2008 guidelines [77]. Additionally, appetite and caloric intake are influenced by factors such as vomiting, gastroesophageal reflux, oral aversion, delayed gastric emptying, nausea, high cytokine levels, renal tubular acidosis and levels of ghrelin and leptin hormone and must be taken into consideration [76]. According to KDOQI guidelines, in addition to iron supplementation, folic acid and vitamin B12 supplementation may be included in the management of anemia [77]. Canepa et al., estimate that up to 40% of children with CKD suffer from hyperhomocysteinemia [78], which is strongly associated with arterial stiffness and the pathogenesis of hypertension [79]. With reference to calcium intake, current KDOQI guidelines recommend that these patients should intake between 100% and 200% of the daily recommended value and that the calculation of this value must account for calcium-based phosphate binders [8]. The form of calcium supplementation must be considered carefully, as calcium chloride may exacerbate metabolic acidosis, while calcium citrate may contribute to aluminum toxicity [76]. KDOQI provides specific values for daily recommended protein intake for children with CKD stages 3 to 5 and 5D and individualizes said recommendations based on patient age and RRT modality [77]. For children on HD, their guidelines suggest 0.1 g/kg/d more than the standard DRI to compensate for dialytic losses, while for those on PD, an intake of 0.15–0.3 g/kg/d over the DRI is recommended [77]. Finally, in children who must restrict sodium intake, KDOQI guidelines recommend <1500/2400 mg/day [77]. It is evident that the maintenance of adequate nutritional status in the pediatric CKD group, especially those treated with PD, poses a unique challenge. Vigilant assessment and adjustment throughout the growth period must be made under the watchful eye of a renal dietician in order to prevent detrimental outcomes [76].

## 8. Nutritional Guidelines for PD Patients

Protein is a macronutrient especially important amongst peritoneal dialysis patients owing to the protein wasting state experienced during dialysis; as such, further investigation into which protein sources are most beneficial to PD patients is highly warranted. The PKD Foundation [7] provides a specific daily recommendation of 1.2–1.4 g/kg/day, which is slightly higher than the European guideline [80] recommendations of 1.0–1.2 g/kg/day. This is coherent with findings by Piriano et al., that PD patients with an intake equal to or greater than 1 g/kg/day remain in a neutral or positive nitrogen balance while those with a lower intake are at risk of being in the negative [2]. KDIGO and Canadian guidelines do not include specific protein intake recommendation [9,81,82]. When comparing the nutritional intake between three large groups: non-dialysis CKD, HD and PD, the only nutrient level intake that differs is protein. Non-dialysis CKD patients require 0.6–0.8 g/kg and 1.0 g/kg/day during illness, while HD patients require >1.2 g/kg/day and PD patients require 1.2 g/kg/day. One scenario in which PD patients require higher protein intake is during peritonitis, and in such cases, the requirement was >1.5 g/kg/day [83].

The PKD Foundation recommends a daily intake of 1000 mg of calcium, similar to KDIGO’s recommendations of 1000–1200 g [7,81] The PKD Foundation did not set an upper limit with regards to potassium intake. In contrast, recommendations by the National Kidney Foundation state that patients should follow a potassium-restricted diet with no more than 2500 mg daily, as serum values of 5.1 or higher may induce arrhythmias and lead to myocardial infarctions [8]. The National Kidney Foundation does note the impact of high phosphorus levels on bone density and recommends supplementation with phosphate binders as a means of avoiding the detrimental consequences of hyperphosphatemia [8]. KDIGO guidelines concerning dietary phosphorus state that patients should not exceed a daily intake of 4000 mg. Daily sodium intake is limited to 3000 mg as per PKD Foundation recommendations [7]. This must be strictly monitored in patients with CKD, and further limitations should be placed on patients in later stages of the disease where the risk of complications such as heart failure increases.

The National Kidney Foundation offers free brochures with general guidelines for patients on PD, but these recommendations are very vague and many micro- and macro-nutrient groups lack specific values [8]. Not all PD patients have access to dietician services, and not all centers are able to provide patients with dietician counseling at the start of treatment. Troublingly, Amalia et al., published that the majority of surveyed PD patients reported salt intake exceeding recommended levels [84]. This emphasizes that information and reliable guidelines are important and should be made readily available to the growing PD patient population. Furthermore, it is crucial that more research be conducted in this field with the goal of ultimately improving health outcomes.

## 9. Adjuvant Therapies

As plant-based diets increase in popularity, it may be worth establishing the forms of protein supplementation (animal-based versus plant-based) that result in the most favorable outcomes for PD patients. Research by Cases et al., suggests that an increased proportion of protein intake from plant-based sources is associated with lower mortality in CKD [85]. Although vegetable-based diets increase potentially harmful potassium levels, there are clear benefits, with effective decrease in blood pressure, prevention of metabolic disease and delay in progression of CKD [85]. During a 13-month period, patients suffering from stage 3–4 CKD who followed a predetermined vegan diet consisting of legumes and cereals did not suffer from nutritional deficiencies. The authors suggest that such diets may be an affordable and appetizing alternative to the low protein diets that are often prescribed to this patient population [85] and challenge the common belief that a plant-based diet provides insufficient levels of amino acids and nutrients. Nutritional imbalances as a result of PD may be alternatively corrected through physical activity, with increased levels of exercise reported to improve body composition [86]. Elderly patients on PD report a high prevalence of low-performance capacity [86], a consequence of age as well as malnutrition and inflammation [86]. Cupusti et al., suggest regular evaluation of physical activity levels through the use of the Rapid Assessment of Physical Activity (RAPA) and the 30” Sit-to-Stand (STS) test [86]. This is to aid ready recognition of impaired ability and level of physical activity and allow for effective implementation of exercise programs [86]. Another area of interest is related to endocrinologic therapy. The use of daily ghrelin has been shown to improve appetite as well as increase energy intake in patients [87,88]. Furthermore, there was continued responsiveness after several consecutive days of therapy, alluding to the fact that such therapy could be used long term for appetite stimulation [87,88] Cachectic patients have benefited from megestrol acetate (MA) therapy in a randomized control study conducted by Yeh et al., PD patients treated with oral MA were shown to improve body weight parameters and the ability to exercise [89]. Although MA is a strong orexigenic agent, its use has not been associated with lowered mortality [90]

## 10. Summary

Nutritional intake and status in peritoneal dialysis patients may prove challenging to assess and correct. Evaluating patients frequently and regularly not only ensures patient education but maintains adequate macro- and micronutrient intake for reduction in morbidity and mortality, as well as improvement in quality of life. A multidisciplinary team with a renal dietician may help to address issues related to inadequate patient knowledge. Further research regarding nutrition must be undertaken to help improve health outcomes in peritoneal dialysis patients.

## Figures and Tables

**Table 1:** Daily macro- and micro-nutrient intake.

	Protein	Calcium	Phosphorus	Sodium	Potassium
PDK	1.2–1.4 g/kg	1000 mg	≥1200 mg	2000–3000 mg	None
KDIGO	None	1000–1200 mg	<4000 mg	None	
Europe	≥1.2 g/kg	None			
Canada	None				

KDIGO—Kidney Disease Improving Global Outcome, UTL—Upper tolerable limit, PKD—Polycystic Kidney Disease Foundation.

**Table 2:** Global Nutritional Recommendation in PD.

Society	Recommendation
KDIGO [7,8]	Restriction of dietary calcium binders in all stages of chronic kidney disease without limitations
	Phytate consumption: anti-oxidant and anti-cancer properties, ability to hinder nutrient absorption
	Importance of minimizing hyperphosphatemia in patients through the ingestion of food additives to an upper tolerable limit of 4000 mg (3000 in those >70 years of age)
	Lack of data which associates dietary restrictions to improved outcomes in patients with stage G3A-G4 chronic renal disease
PKD Foundation [7]	Maintaining blood calcium levels between 8.4 and 9.5 mg/dL
	Phosphorus in the diet may be restricted if blood levels reach greater than 5.0 mg/dL
National Kidney Foundation [8]	Phosphorus in the diet may be restricted if blood levels reach greater than 5.0 mg/dL
Canadian Guidelines [9]	Reduce levels of sodium, phosphorus and fluids
	Increase intake of potassium and protein
	Patient’s following a potassium restricted diet should limit their intake to 2000 milligrams per day, in constant to the standard recommendation of 3500 to 4500 milligrams daily for healthy patients
